# Contrast-Enhanced Ultrasonography Enables the Detection of a Cold Pressor Test-Induced Increase in Renal Microcirculation in Healthy Participants

**DOI:** 10.3389/fcvm.2022.899327

**Published:** 2022-05-20

**Authors:** Mariëlle C. Hendriks-Balk, Aikaterini Damianaki, Erietta Polychronopoulou, Wendy Brito, Menno Pruijm, Gregoire Wuerzner

**Affiliations:** Service of Nephrology and Hypertension, Lausanne University Hospital and University of Lausanne, Lausanne, Switzerland

**Keywords:** contrast-enhanced ultrasound (CEUS), cold pressor test, blood pressure, renal perfusion, renal resistive index (RRI), microcirculation

## Abstract

**Background:**

Renal microcirculation is essential for regulation of the glomerular filtration rate, the reabsorption of salt and water from the interstitium, and hence the blood pressure. Renal ultrasonography coupled to Doppler analysis and contrast-enhanced ultrasound enables the study of renal perfusion. So far, physiologic interventions have rarely been performed to assess the renal perfusion. The objective of our study was to measure the renal perfusion in response to a cold pressor test (CPT).

**Methods:**

Healthy adult participants were exposed to a 2 min CPT or a sham exposure (body temperature). Systemic hemodynamics, renal resistive index (RRI) and renal perfusion index (PI) were measured before and during the CPT or the sham exposure. Renal responses were compared using a paired Student's *t*-test or Wilcoxon signed rank test. Pearson correlation test was used to test association of variables of interest.

**Results:**

Forty-one normotensive participants (21 women) were included in the study. Mean blood pressure and heart rate both increased with the CPT. The RRI decreased from 0.60 ± 0.05 arbitrary units (AU) to 0.58 ± 0.05 AU (*p* < 0.05) and the PI increased from 2,074 AU (1,358–3,346) to 3,800 AU (2,118–6,399) (*p* < 0.05) (+66% (24–106%)). Compared to the sham exposure, the increase in PI with the CPT was more marked. There was a negative association between the increase in heart rate and mean blood pressure with the RRI (*r*: −0.550, *p* = 0.002 and *r*: −0.395, *P* = 0.016), respectively.

**Conclusion:**

Doppler Ultrasound and CEUS enable the detection of physiological changes within the macro- and microvascular renal circulation. The CPT decreases the RRI and increases the PI. Whether these changes are present in pathological states such as diabetes or hypertension will need additional studies.

## Introduction

Renal ultrasonography is a non-invasive, non-radiating, broadly accessible and well-tolerated imaging technique, which has been used in clinical practice to assess kidney anatomy for more than 40 years (1). Coupled with Doppler effect and ultrasound contrast agents, it also allows the assessment of the renal macro- and microcirculation (2, 3). Its evaluation is important since the kidney receives 20% of the cardiac output and the renal perfusion is essential for normal kidney function. Moreover, disturbance in blood flow is among the main mechanisms of acute and chronic kidney injury. Renal perfusion is controlled by many systems, including renal autoregulation, the renin-angiotensin system and the sympathetic nervous system. The latter leads to a reduction in global renal blood flow, but its effect on the microcirculation in the kidney is not fully understood.

Contrast-enhanced ultrasonography (CEUS) uses the interaction of the ultrasound beam with gas-filled microbubbles in suspension (4). These microbubbles have a mean diameter range of 1.5–2.5 μm, small enough to move into the capillaries but large enough to stay in the vascular system. Nowadays, CEUS is approved during echocardiography, for the characterization of focal liver lesions and the evaluation of vesicoureteral reflux with CEUS in adult and pediatric patients. CEUS has emerged as a valuable and safe imaging technology complementing and enhancing standard ultrasonography in a variety of organs including the liver, the bladder, the kidneys, the urinary tract, the spleen and the skeletal muscles as well as in tumors (5). The ultrasound contrast agents are safe, without nephrotoxicity or hepatotoxicity, and easily administered at the bedside, which explain their potential wide-range use.

Although CEUS is in theory an ideal tool to detect time- or intervention-dependent changes of the renal hemodynamics, only a few studies mention the use of CEUS to quantify renal microperfusion or renal blood flow (2, 6–8). So far, changes in microcirculation have been detected in humans after the infusion of noradrenaline, angiotensin II or after a high protein meal (2, 8, 9). Changes in renal microperfusion secondary to a physiologic stress test such as the cold pressor test (CPT) have not been studied yet and could prove valuable in patients in whom pathophysiologic changes are expected. Therefore, the aim of this study was to assess whether CEUS could detect differences in renal cortical microcirculation during a CPT.

## Methods

### Study Population

Adult healthy normotensive participants without any drug abuse and/or consumption were eligible to participate in the study. Participants with prior allergic reaction to the contrast agent SonoVue®, with ongoing pregnancy, or taking drugs affecting renal hemodynamics such as non-steroidal anti-inflammatory drugs or vaso-active drugs, were excluded from the study.

### Study Design and Settings

This study was part of a prospective single-center sham-controlled study evaluating the effect of a CPT on brain and kidney function. Participants abstained from smoking and alcohol intake for 48 h and strenuous physical activity for 24 h before the study day. Caffeine-containing products, energy drinks, chocolate and bananas were prohibited from 6 pm the day before the study. On the study day, fasting participants arrived at the investigation center between 8h00 and 10h00. A venous catheter was inserted in one arm to allow blood samples to be taken as well as the intravenous administration of the contrast-agent (Sonovue®, Bracco Internationa B.V, Amsterdam, The Netherlands).

Blood pressure and heart rate were measured continuously using the Finapres® NOVA (Finapres Medical Systems, Enschede, The Netherlands) throughout the experiment. The Finapres® NOVA also measured total peripheral resistance (TPR) and cardiac output (CO). After 5 min of signal stabilization, the finger blood pressure was calibrated with a brachial oscillometric blood pressure measurement. This calibration was repeated at least 2 min before each CPT.

**Figure 1:**
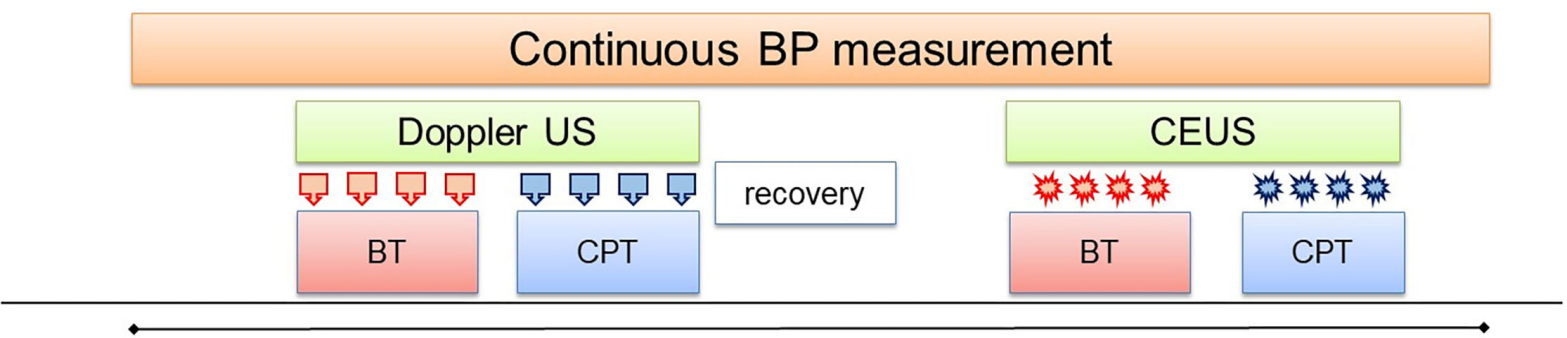
Study design. Both feet were placed in a water bath initially filled with water at body temperature (BT) for baseline Doppler US measurements (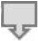). Thereafter, the water was replaced by cold water (2–4°C) for 2 min for the measurements during the CPT. In the second phase, the same procedure was repeated for the CEUS measurements (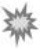).

The modified CPT test consisted in placing both feet in a footbath filled with water at body temperature (35–37°C, sham) for baseline BP and ultrasound measurements. The water was replaced by cold water (2–4°C) for 2 min during which systemic hemodynamic and renal ultrasound measurements were repeated. Thereafter, the cold water was substituted by water at body temperature (recovery phase). This procedure was conducted twice, with at least 10 min between the two phases. During the first phase, Doppler ultrasound was performed before and during the CPT in order to assess changes in intra-renal macrocirculation. During the second phase, CEUS was performed before and during the CPT in order to assess changes in renal microcirculation (see Figure 1).

Baseline systemic hemodynamic variables (systolic blood pressure: SBP, diastolic blood pressure: DBP, mean blood pressure: MBP and heart rate: HR) were calculated as the average of 2 min continuous recording before CPT. During CPT, the systemic hemodynamic variables were calculated as the mean of continuous recording during CPT (up to 2 min). Maximal CPT systemic hemodynamic variables were calculated as the mean of the last 30 s of the CPT. Recovery systemic hemodynamic variables were calculated as the mean of the last minute of the 2 min recovery period after the CPT.

### Renal Ultrasound Measurements

We used a Samsung RS80A ultrasound device and a dedicated abdominal probe (3–5 MHz) for all measurements. All exams were performed by the same experienced sonographer (WB). The kidneys were initially examined in B-mode. In case of anatomic abnormalities such as cysts, tumors or hydronephrosis, subjects were excluded from the study. Next, Doppler mode was applied in order to select the segmental artery with the easiest accessibility and highest quality of Doppler waveforms. For this reason, in almost all participants, the right kidney was chosen to determine the renal resistive index (RRI). Measurements of RRI were performed in expiratory breath-hold and were repeated at least 4 times at baseline and during the CPT. Special care was taken to select the same segmental artery at each measurement. The RRI was calculated as (peak systolic velocity—end diastolic velocity)/peak systolic velocity in the color Doppler ultrasound mode.

For the CEUS exam, the probe was oriented in such a way that an optimal long axis view of the right kidney was obtained, in order to assure a large cortical surface area. The Sonovue® contrast agent was injected as a continuous infusion with a special rotating syringe pump (Vueject®, Bracco SA, Milano, Italy) at a continuous rate of ~1 ml/min (0.015 ml/kg/min for a person of 70 kg). Image depth, focus, gain and frame rate were optimized and held constant during the experiment. To quantify intra-renal perfusion the destruction-replenishment technique was used as described previously (2, 7).

Four consecutive destruction-replenishment sequences were performed during each condition after about 60–90 s of microbubbles infusion, required to reach a steady state and intra-renal saturation. All destruction-replenishment sequences were performed in breath hold in order to avoid large movement artifacts. Destruction was obtained by applying a flash of increased ultrasound intensity [five pulses with high mechanical index (MI 1.4)] and replenishment was observed at low intensity (MI 0.08).

Images were exported in digital imaging and communication in medicine (DICOM) format and analyzed with the dedicated software Vuebox® (Bracco Research, Geneva, Switzerland) using the replenishment model. Regions of interest (ROI) were drawn manually and included the largest part of the renal cortex, as published previously (6). Compensation of movement (due to minor breathing artifacts or small movements of the probe) was applied to all sequences before analysis. The DICOM data in each ROI were converted into echo-power data that are directly proportional to the concentration of the contrast-agent.

For each ROI the software generates a time-intensity curve (intensity of replenishment over time) and calculates the relative blood volume (rBV), the mean transit time (mTT) and the perfusion index (PI; PI = rBV/mTT) which is proportional to the blood flow (10, 11). The rBV [expressed in arbitrary units (a.u.)] is a measure of the maximal intensity after complete replenishment. The mTT (seconds) is the time needed after microbubble destruction to reach 50% of the maximal intensity signal (point at maximum slope or wash-in rate) (6). Only sequences with a quality of fit of more than 85% between the echo-power signal and the time-intensity curve in a specific ROI were used.

### Data and Statistical Analysis

Study data were captured and managed using REDCap electronic data capture tools hosted at CHUV (12, 13).

Data are expressed as means ± standard deviation (SD) or as median (interquartile range), depending on normality of distribution. In case of normal distribution, a paired Student's *t*-test was used to test for significant differences between variables captured during baseline and the CPT. A Wilcoxon signed rank test was used when data were not normally distributed. A two-sided *p*-value <0.05 was considered statistically significant. Linear correlations between systemic and renal hemodynamics were measured using the Pearson correlation coefficient. Dependency on age or gender were verified using linear regression. All statistical analyses were performed with Stata 16 (Statacorp, College Station, TX, USA).

## Results

### Participants

We included 41 normotensive healthy participants (21 women). Their mean age was 34 ± 10 years and their mean BMI was 23.0 ± 2.5 kg/m^2^. Of these participants, 19 (9 women) participated to the sham stimulation. The CPT was well tolerated in all participants except one, who reported transient chest pain. The pain spontaneously resolved with seconds. No changes in ECG and in plasma troponin-t or CK enzymes were detected. Pain rating of the cold sensation was 5.9 ± 2.3 on a scale of 10, indicating a moderate pain sensation. Echography quality of one male participant was insufficient to be analyzed. For one female participant it was not possible to insert a catheter for the intravenous administration of the contrast-agent for CEUS.

### Effect of CPT on Systemic Hemodynamics

**Figure 2:**
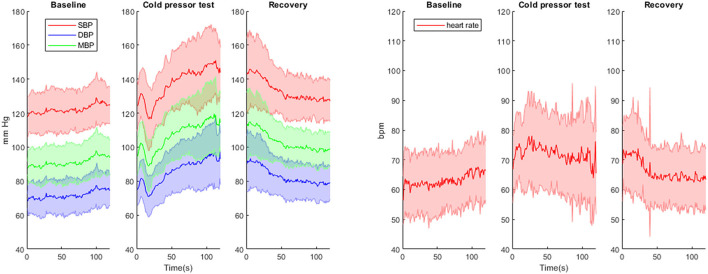
Continuous blood pressure (mean +/-SD) and heart rate (mean +/-SD) measurements during baseline, cold pressor test and recovery. SBP, systolic blood pressure; DBP, diastolic blood pressure; MBP, mean blood pressure.

SBP, DBP, MBP and HR all increased during CPT compared to baseline values (Table 1; Figure 2). After a biphasic response (increase/decrease) in the first 20 s, BP steadily increased and reached maximal levels in the last 30 s of the CPT. HR increased rapidly after the onset of CPT and reached maximal values within the first 30 s. BP values and HR decreased rapidly in the recovery period after the CPT (Table 1; Figure 2). Total peripheral resistance also increased during CPT and remained increased during the 2 min in the recovery period. Cardiac output increased during the first 30 s of the CPT and returned to baseline before the end of the CPT (Table 1).

**Table 1:** Systemic hemodynamic during baseline, CPT, maximal CPT (last 30 s of CPT) and recovery (last 60 s).

	**Baseline**	**CPT**	**maxCPT**	**Recovery**
SBP (mm Hg)	122.5 ± 10.9	135.5 ± 14.2^**^	144.4 ± 19.3^**^	128.2 ± 11.0^**^
DBP (mm Hg)	71.8 ± 9.3	84.1 ± 11.6^**^	90.5 ± 15.0^**^	78.4 ± 9.8^**^
MBP (mm Hg)	91.3 ± 10.3	105.1 ± 13.5^**^	112.5 ± 17.6^**^	98.2 ± 9.7^**^
HR (bpm)	63.0 ± 8.8	72.8 ± 11.8^**^	70.2 ± 12.3^**^	63.0 ± 9.3
CO (l/min)	5.00 ± 0.98	5.41 ± 1.36^**^	5.09 ± 1.30	4.76 ± 1.08^**^
TPR (mm Hg^*^s/ml)	1.17 ± 0.41	1.27 ± 0.57^*^	1.35 ± 0.48^**^	1.36 ± 0.62^**^

*Data are presented as mean ± SD*.

* *p < 0.01*,

** *p < 0.001 compared to baseline, n = 40*.

*CPT, cold pressor test; SBP, systolic blood pressure; DBP, diastolic blood pressure; MBP, mean blood pressure; HR, heart rate; CO, cardiac output; TPR, total peripheral resistance*.

### Effect of CPT on Renal Hemodynamics

**Figure 3:**
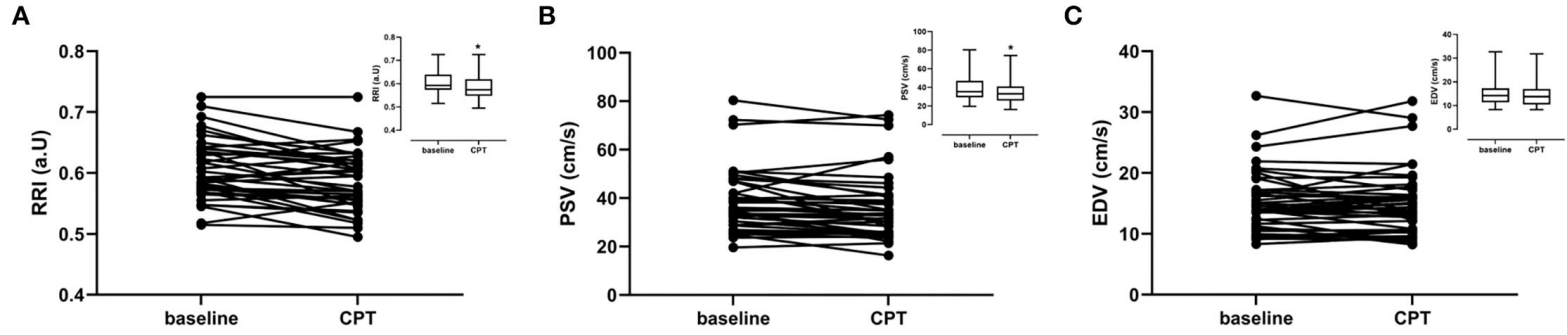
Renal ultrasound parameters per subject for baseline and cold pressor test (CPT). Graph inserts are the mean values ± SD. **(A)** Renal resistivity index (RRI) **(B)** Peak Systolic Velocity (PSV). **(C)** End diastolic velocity (EDV).

During CPT the RRI decreased by 0.02 ± 0.03 (−3.3%). This lower RRI was driven by the decrease in peak systolic velocity observed during the CPT. The end diastolic velocity was not affected by the CPT (Table 2; Figure 3).

**Table 2:** Renal hemodynamic during baseline and CPT.

	**Baseline**	**CPT**	***p*-value**
***Doppler US (n = 40):***			
RRI (a.u.)	0.60 ± 0.05	0.58 ± 0.05	0.0001
PSV (cm/s)	39.0± 13.2	36.3 ± 13.6	0.0059
EDV (cm/s)	15.3 ± 5.1	14.9 ± 5.5	0.4358
***CEUS (n = 39):***			
PI (a.u.)	2,074 (1,358–3,346)	3,800 (2,118–6,399)	<0.0001
rBV (a.u.)	4,195 (2,545–7,172)	6,722 (4,303–9,351)	<0.0001
mTT (s)	1.98 (1.39–2.73)	1.71 (1.42–2.29)	0.0052

*Data are presented as mean ± SD or median (interquartile range)*.

*CPT, cold pressor test; RRI, renal resistive index; PSV, peak systolic velocity; EDV, end diastolic velocity; PI, perfusion index; rBV, relative blood volume; mTT, mean transit time*.

**Figure 4:**
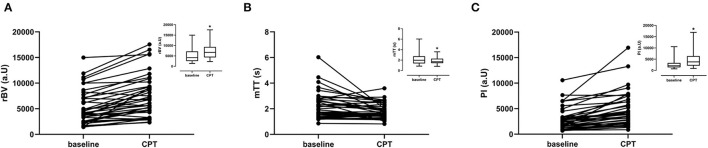
CEUS parameters per subject for baseline and CPT. Graph inserts are the mean values ± SD. **(A)** Relative blood volume (rBV). **(B)** Mean transit time (mTT). **(C)** Perfusion index (PI).

The cortical rBV increased while mTT decreased during CPT (Table 2; Figure 4). Consequently, the perfusion index increased from 2,074 (1,358–3,346) to 3,800 (2,118–6,399) corresponding to a median increase of +66% (24–106%). The increase in PI and rBV and the decrease in mTT were not different between men and women.

**Figure 5:**
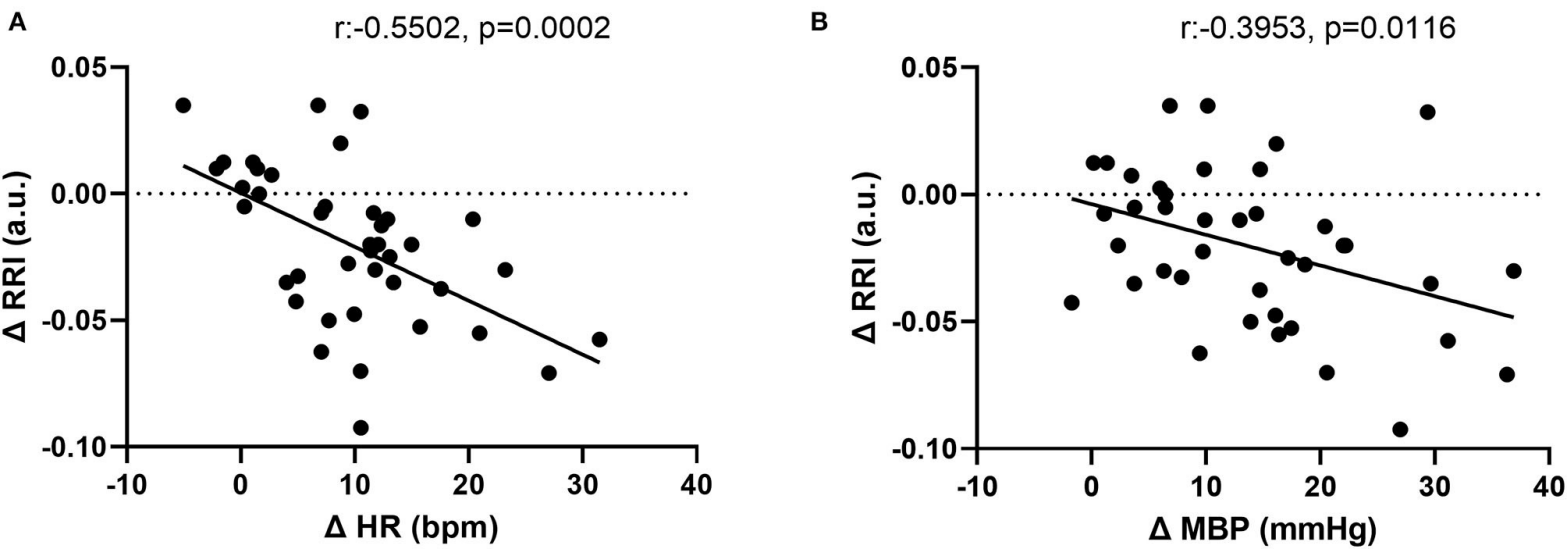
Correlation between changes in **(A)** renal resistive index (RRI) and heart rate (HR). **(B)** RRI and mean blood pressure (MBP).

During the sham stimulation the RRI, peak systolic velocity, end diastolic velocity did not change (Table 3; Figure 5). However, PI slightly increased (from 1,750 (1,050–3,620) during baseline to 3,000 (1,360–5,040) during sham stimulation), which is mainly driven by an increase in relative blood volume [from 3,310 (1,730–6,700) to 4,870 (2,860–8,250)]. The mean transit time decreased [from 1.90 (1.33–2.12) at baseline to 1.77(1.34–2.11)] (Table 3). These results indicate a time-dependent effect of the microbubble intensity/luminosity. However, this increase in perfusion index in the sham stimulation [delta +876 (383–1280)] was less than the increase of the PI during the CPT [delta +1600 (616–3,240), *p* = 0.0082] shown in Figure 5.

**Table 3:** Renal hemodynamic during baseline and sham stimulation compared to CPT stimulation.

	**Sham baseline**	**Sham stimulation**	**Delta**	**Baseline**	**CPT**	**Delta**
***Doppler US:***						
RRI (a.u.)	0.61 ± 0.06	0.61 ± 0.05	−0.004 ± 0.02	0.62 ± 0.05	0.60 ± 0.05^**^	−0.02 ± 0.02
PSV (cm/s)	38.49 ± 11.65	37.28 ± 10.85	−1.21 ± 3.48	38.66 ± 15.16	36.07 ± 14.87^**^	−2.59 ± 4.67
EDV (cm/s)	14.77 ± 5.35	14.55 ± 4.97	−0.21 ± 1.39	14.82 ± 6.15	14.43 ± 5.83	−0.39 ± 2.10
***CEUS:***						
PI (a.u.)	1,752 (1,050–3,619)	3,001 (1,361–5,039)^*^	+40 (18 to 81)%	1,963 (1,313–2,829)	3,562 (2,172–6,399)^**^	+79 (42 to 106)%^†^
rBV (a.u.)	3,311 (1,728–6,695)	4,872 (2,864–8,251)^*^	+39 (23 to 65)%	4,103 (2,534–7,019)	6,111 (3,262–9,351)^**^	+45 (30 to 55)%
mTT (s)	1.90 (1.33–2.12)	1.77 (1.34–2.11)	+1 (−15 to 23)%	2.23 (1.68–2.99)	1.62 (1.39–2.29)^**^	−18 (−39 to−1)%^†^

*Data are presented as mean ± SD or median (interquartile range)*.

*n = 19*,

* *p < 0.05 from sham baseline*,

** *p < 0.05 from baseline*,

† *p < 0.05 from delta sham stimulation*.

*RRI, renal resistive index; PSV, peak systolic velocity; EDV, end diastolic velocity; PI, perfusion index; rBV, relative blood volume; mTT, mean transit time*.

### Correlation Between Systemic and Renal Hemodynamic Changes Induced by CPT

The CPT-induced increase in HR correlated inversely with the change in RRI (r = −0.55 p = 0.0002). In addition, the CPT-induced an increase in MBP, which was also inversely associated with the change in RRI (−0.40 (*p* = 0.0116)) (Figure 5). These associations were independent from age and sex.

Although there seemed to be an association between the increase in PI and the increase in blood pressure [*r* = 0.38 *p* = 0.0178 (SBP), *r* = 0.37 *p* = 0.0236 (DBP), *r* = 0.37 *p* = 0.0209 (MBP)] this correlation was secondary to the results of one subject (outlier). The association between the increase in PI and the increase in HR (*r* = 0.25 *p* = 0.1323) was also driven by one outlier. No correlation was found between the increase in cardiac output and the increase in PI (*r* = 0.14 *p* = 0.39). Age and sex had no influence on the correlations.

## Discussion

Renal microcirculation plays a major role in the oxygen supply, plasma filtration and the exchange of electrolytes and water. To our knowledge, this is the first demonstration that physiological changes in circulation induced by the CPT are detectable both by Doppler ultrasound and by CEUS. In the segmental arteries of the kidney, the CPT decreased the RRI, which was driven by a decrease in the peak systolic velocity. This decrease in RRI correlated well with the increases in systemic parameters heart rate and MBP during the CPT. In the microvasculature of the renal cortex the CPT induced an increase in the PI by increasing the relative blood volume and decreasing the mean transit time. A clear association with heart and MBP was not found with the PI.

The increase in blood pressure and heart rate in response to CPT is in line with several previous reports (14, 15). The CPT is known to activate the sympathetic nervous system with peripheral arteriolar vasoconstriction as a predominant response, which in turn results in an increase in blood pressure (15–17). Although vasoconstriction might be the predominant systemic response to cold stress, it can be affected by several factors such as age, type of cold exposure (whole body skin-surface, face, hand or foot), duration of cold exposure and health status (18, 19). In addition, different vascular beds may react differentially to a cold stress.

The decrease in RRI and the increase in PI in the renal cortex during CPT in our study on healthy normotensive subjects suggest rather a vasodilation of the renal vasculature than a vasoconstriction in response to CPT despite of the observed increase in total peripheral resistance. This CPT-induced vasodilation has been reported in other organs. Indeed, normal coronary arteries were shown to dilate in response to CPT, whereas in atherosclerotic coronary arteries the CPT caused a vasoconstriction (20, 21). The vasodilation in normal coronary arteries in response to CPT possibly reflects a higher myocardial oxygen demand due to the rise in HR and SBP and hence an increase in coronary blood flow. Such a mechanism is unlikely in the kidneys, as renal tissue oxygenation will not increase when renal blood flow increases; the latter will increase GFR and metabolic work load in order to reabsorb filtered electrolytes and other substances (22).

Lafleche et al. reported an increase in carotid artery diameter and brachial artery diameter in normotensive subjects in response to a CPT on the hand, suggesting again local vasodilation (23). However, in borderline hypertensive subjects the diameter of the carotid artery decreased during CPT whereas there was still an increase in brachial artery diameter. Another study described differential responses of the common carotid artery compared to the internal carotid artery during CPT applied to the foot in young individuals (24). The diameter of the common carotid artery increased during CPT, whereas the diameter of the internal carotid artery did not change. In both cases blood flow increased but blood velocity did not change. In older individuals, the increase in common carotid artery during CPT was blunted (24). These data suggest that the location and extent of exposure to cold may play a role. In this context, whole body skin-surface cooling decreases vascular conductance of skin, brachial, celiac, superior mesenteric and renal arteries suggesting vasoconstriction in response to cold exposure (16). An explanation might be that the whole body skin-surface cooling increases the skin sympathetic nerve activity but not the muscle sympathetic nerve activity (25, 26) whereas CPT induces muscle sympathetic nerve activity but not skin sympathetic activity (15, 27).

Taken together, we hypothesize that in healthy participants the CPT applied at the feet activated essentially the muscle sympathetic nerve activity, which favors vasodilation of renal and possibly coronary and carotid arteries, whereas peripheral vessels constrict. As we did not measure nerve activity nor the diameter of the arteries, this remains purely hypothetical. However, this hypothesis of CPT causing renal vasodilation resulting in increases in PI is in line with a study done by Schneider et al. (2). In their study, they used a pharmacological stress to evaluate changes in the renal microcirculation and showed that Angiotensin II-induced vasoconstriction caused a decrease in the renal PI by 47 to 65% depending on the dose and vasodilation by Captopril an increase in PI by 35%. This vasodilation-induced increase in PI is lower than the effect of CPT in our study, which caused an increase in PI of 66%. However, the stronger effect of CPT on PI observed in our study can partly be explained by the increase in PI in time as seen in the sham stimulation. To the best of our knowledge, our study is the first to study a sham-procedure in CEUS-assessed measurement of microbubbles to detect any time effect of PI. Therefore, we cannot judge whether the lack of tissue saturation, respectively, diluted or reinforced the effects of vasoconstrictive or vasodilative drugs such as angiotensin-II, noradrenaline or ACE-inhibitors in previous studies (2, 9). Nevertheless, part of the increase in PI is secondary to the CPT itself and goes in the same direction of the vasodilation-induced change in PI, suggesting CPT induces a vasodilation in the renal microvasculature.

It is important to differentiate the CPT from other techniques that have been used to stimulate the sympathetic nervous system, such as the handgrip test. In an elegant study, Haddock et al. (28) showed that the handgrip exercise induced a decrease in renal arterial flow as well as a decrease in perfusion in the renal cortex and medulla. The handgrip test is an exercise stress test in contrast to the CPT, which is a non-exercise generalized stress test. An exercise-based stress tests involves muscle activation and, hence, activate the muscle metaboreflex and mechanoreflex, which may play a role in the stress response. The difference between the effect of the handgrip test on renal perfusion in their study and the CPT effect in our study might be explained by the different underlying mechanisms to activate the sympathetic nervous system.

Vasoconstriction of peripheral vessels leads to in an increase in systemic vascular resistance, hence an increase in renal vascular resistance, which in many papers is wrongly used interchangeably with the RRI. The RRI is assumed to reflect several renal vascular properties like renal vascular resistance, vascular compliance, interstitial and venous pressures. It is therefore used as a marker for kidney damage in a variety of clinical conditions from renal vascular disease to chronic kidney disease and renal transplant, but also in diabetes and hypertension. However, increasing evidence has shown that systemic vascular properties such as pulse pressure, blood pressure, heart rate, arterial stiffness, are also important factors that influence the RRI (3, 29–32). This is not surprising when taking into account that the kidney receives about 20% of the cardiac output although it represents only 1% of the body weight (33). The RRI is therefore dependent of a complex interaction between all these factors, which might include compensation mechanisms as well.

In our study on healthy normotensive subjects, the decrease in RRI in response to CPT is correlated to the increase in heart rate and the increase in blood pressure. By artificially increasing the heart rate Mostbeck et al. (34) showed a decrease in RRI and he proposed that RRI should be corrected for heart rate using a specific regression equation. In animal studies using an isolated kidney a linear relationship was found between the pulse pressure index [(SBP-DBP)/SBP] and the RRI but no direct relation between renal vascular resistance and RRI (35).

The increase in the renal PI in response to CPT doesn't seem to be correlated with the increase in blood pressure and the increase in heart rate. This is in line with previous studies. Schneider et al. (2) showed that vasoconstriction induced by Angiotensin II in healthy subjects caused an increase in mean blood pressure but a decrease in the renal cortical PI as measured by CEUS. In another study on critically ill patients, his group observed that a noradrenaline-induced increased in BP was not correlated with an increase in renal cortical perfusion (7). Those studies indicate that there is little or no correlation between changes in BP and changes in renal cortical microcirculation when a pharmacological stimulus is used.

Our study has some limitations and shows several confounding factors that have to be taken into account for future research. In our study CEUS was always measured after Doppler US, hence a habituation effect to the CPT could have affected the CEUS responses. However, the blood pressure response was constant with each stimulation. To minimize heterogeneity of measurements within subjects we used the complete visible renal cortical region as ROI. However, it is possible that subjects reacted to the CPT in such a way that the kidney position changed and less renal cortex was visible and hence the ROI slightly different from the baseline.

The inter-individual variability in CEUS signal intensity at baseline was high, as well as the response to CPT. In addition, the results of the sham stimulation show that CEUS signal intensity (i.e., renal blood volume) tends to increase in time even after the fourth measurement. In our opinion, the most likely explanation for this increase in CEUS signal intensity is that steady state was not reached after 1–1.5 min of perfusion before starting the destruction-replenishment sequences. Theoretically, the breath hold technique may have delayed the natural elimination of the microbubbles, as their sulfur hexafluoride content is exhaled by the lungs. However, we believe that the breath holds were too short (duration: 6–8 s) to have a significant impact on their elimination. In line with this hypothesis, in a previous study by our group applying a similar protocol we also observed that PI increased between the second and fourth minute of perfusion, although only half the number of apneas were performed in that study (6). Besides, prolonged breath hold is expected to reduce, not increase, renal perfusion. This was demonstrated in an elegant study by elite divers who performed breath holds up to 2 min during MRI scans (36). Ideally, a longer period of continuous perfusion should probably be applied in order to assure tissue saturation with microbubbles. However, this would roughly double the costs of the already expensive microbubbles. Hence, further research is needed to establish the perfect perfusion protocol.

## Conclusion

This study demonstrates that the CPT combined to Doppler Ultrasound and CEUS can be used to evaluate the renal macro- and microcirculatory responses. In healthy subjects, the RRI was decreased and the renal cortical PI increased upon CPT, suggesting renal vasodilation, whereas BP levels, estimated cardiac output and estimated total peripheral resistance were increased suggesting a systemic vasoconstriction. Whether the renal microcirculation response to a CPT in pathologic states such as diabetes or hypertension differ from healthy volunteers will need future studies.

## Data Availability Statement

The raw data supporting the conclusions of this article will be made available by the authors, without undue reservation.

## Ethics Statement

The study was approved by the local Ethics Committee “Commission cantonale d'éthique de la recherche sur l'être humain” (www.cer-vd.ch) and was carried out in accordance with the principles of the Declaration of Helsinki. All subjects provided written informed consent prior to inclusion. The study is registered at clinicaltrials.gov (NCT03473275). The participants provided their written informed consent to participate in this study.

## Author Contributions

MH-B and GW designed the study, analyzed the data, and wrote the manuscript. AD and EP took care of the participants and captured the data. WB performed the ultrasonography. MP supervised ultrasonography and reviewed the manuscript. All authors contributed to the article and approved the submitted version.

## Funding

This study was supported by grants from the Swiss National Foundation (SNF 320030_179379) and the Swiss Cardiology Foundation. This study also received partial funding from Medtronic Public Limited Company.

## Conflict of Interest

The authors declare that the research was conducted in the absence of any commercial or financial relationships that could be construed as a potential conflict of interest.

## Publisher's Note

All claims expressed in this article are solely those of the authors and do not necessarily represent those of their affiliated organizations, or those of the publisher, the editors and the reviewers. Any product that may be evaluated in this article, or claim that may be made by its manufacturer, is not guaranteed or endorsed by the publisher.
